# Pruritus Vulvæ Relieved by Iodide of Potassium

**Published:** 1881-03

**Authors:** 


Pruritus Vulvæ Relieved by Iodide of Potassium.—At a meeting of the St. Louis Medico-Chirurgical Society Dr. Bryson related the following case: Having under treatment a patient suffering from fistula in ano and urethral stricture, he learned that the man had syphilis, and gave him constitutional treatment with potassium iodide while he was dilating the stricture. Not long after he was called to treat the wife of this patient for most intolerable pruritus vulvæ. The general condition of the woman seemed to be very good, but she had been married seven years without bearing any children, and had once aborted in the third or fourth month of pregnancy. These facts, in connection with his knowledge of the husband's history, led the doctor to suspect a syphilitic taint in the woman; and he prescribed potassium iodide in doses of three grains three times a day, which was gradually increased to ten grains three times a day. No local treatment was used, and in three days the distressing pruritus entirely disappeared. The iodide was continued for some weeks longer with marked improvement of the spirits and health of the patient.

There was no eruption or other lesion characteristic of syphilis apparent about the vulva, and Dr. Bryson considers that the trouble was due to an obscure syphilitic nerve affection.—*St. Louis Courier Medicine.*

